# Reduction of Mosquito Survival in Mice Vaccinated with* Anopheles stephensi* Glucose Transporter

**DOI:** 10.1155/2017/3428186

**Published:** 2017-07-19

**Authors:** J. Couto, S. Antunes, J. Ferrolho, J. de la Fuente, A. Domingos

**Affiliations:** ^1^Global Health and Tropical Medicine, Instituto de Higiene e Medicina, Universidade Nova de Lisboa (GHMT-IHMT-UNL), Rua da Junqueira 100, 1349-008 Lisboa, Portugal; ^2^SaBio, Instituto de Investigación en Recursos Cinegéticos (IREC), CSIC-UCLM-JCCM, Ronda de Toledo s/n, 13005 Ciudad Real, Spain; ^3^Department of Veterinary Pathobiology, Center for Veterinary Health Sciences, Oklahoma State University, Stillwater, OK 74078, USA

## Abstract

Despite the fact that recent efforts to control/eradicate malaria have contributed to a significant decrease in the number of cases and deaths, the disease remains a global health challenge. Vaccines based on mosquito salivary gland antigens are a potential approach for reducing vector populations and malaria parasites. The* Anopheles AGAP007752* gene encodes for a glucose transporter that is upregulated during* Plasmodium* infection, and its knockdown decreases the number of sporozoites in mosquito salivary glands. These results together with the fact that glucose is a vital source of energy suggested that a glucose transporter is a candidate protective antigen for the control of mosquito infestations and* Plasmodium *infection. To address this hypothesis, herein we investigate the effect of mice vaccination with an immunogenic peptide from mosquito glucose transporter on* Anopheles stephensi* fitness and* Plasmodium berghei* infection. We showed that vaccination with a peptide of glucose transporter reduced mosquito survival by 5% when compared to controls. However, the reduction in* Plasmodium* infection was not significant in mosquitoes fed on vaccinated mice. The effect of the peptide vaccination on mosquito survival is important to reduce infestation by malaria vectors. These results support further research on developing glucose transporter-based vaccines to reduce mosquito fitness.

## 1. Introduction

Despite substantial progress in controlling malaria, the disease is still a public health problem in many countries, even with a notorious reduction on malaria cases and mortality rates of 41% and 62%, respectively, between 2000 and 2015 [1]. The Global Technical Strategy for Malaria 2016–2030 defined as primary targets the reduction of the number of cases and deaths globally by at least 90% and the elimination and prevention of reestablishment of malaria from no less than 35 countries [1, 2].

Vaccines constitute the most efficacious intervention towards infectious diseases control. While some blood-stage vaccine candidates are presently under study, the Mosquirix or RTS,S/AS01 was already approved by the European Medicine Agency and is recommended by the WHO for national immunization programs. This vaccine does not confer full protection against* Plasmodium falciparum*, the deadliest parasite causing human malaria, and, as such, it is often used in combination with other control measures, such as bed nets, indoor residual spraying, and antimalarial drugs, depending on disease prevalence and parasite resistance data from each country [3].

Transmission-blocking vaccines are a good example of an alternative/complementary control measure as these vaccines may disrupt the parasite life cycle in the* Anopheles* sp. mosquito. By reducing the number of infectious vectors and parasite reservoirs, the transmission of* Plasmodium* declines into the people community, leading to local group immunity [4, 5]. The first malaria transmission-blocking vaccine were focused on surface antigens of parasite sexual stages but, recently, target antigens of the mosquito have also been investigated [6]. Due to the crucial role in parasite infection,* Anopheles* midgut or salivary gland-specific antigens have been used to interact with specific receptor-ligand peptides that are essential to block parasite invasion or maintenance [7–10].

Membrane transporter proteins are within the top five protein classes against which Food and Drug Administration-approved drugs are developed [11]. These proteins encompass diverse gene families, namely, major facilitator superfamily transporters that allow shuttling of nutrient and metabolites, which can provide a regulated electrochemical gradient essential for mosquito survival [12]. As occurs with facilitated transporters and transmembrane integral proteins, glucose transporters are likely to be glycosylated [13] and this may modulate parasite recognition by the lobes of the mosquito salivary glands [14, 15], leading to parasite adhesion and/or invasion. Furthermore, these proteins allow passive passage of glucose across the cell membrane enabling the required metabolism of* Plasmodium* spp. for maintenance during sporogonic cycle in the vector [16].

The* AGAP007752* gene, which encodes a glucose transporter (GT), is an example whose properties have been previously evaluated [17]. The gene coding for this GT had the highest expression in* Plasmodium*-infected salivary glands of* A. coluzzii* s.s. (*A. gambiae* molecular M form) in both RNA-seq and relative-qPCR assays in comparison with noninfected groups [17], which suggests that the parasite is probably modulating* AGAP007752* expression to overcome invasion and facilitate its maintenance in the target-organs [14, 16, 18, 19]. Moreover, Pinheiro-Silva et al. (2015) showed that a significant reduction of AGAP007752 mRNA levels using interference RNA results in a reduction of the number of sporozoites by 44% in the salivary glands at 18 days after infection [17]. These findings emphasized the importance of* AGAP007752* overexpression during* Plasmodium* infection and its potential as a candidate protective antigen for the development of a vaccine. Considering that malaria is a vector-borne disease a dual-effect vaccine is an attractive solution by targeting both mosquito and pathogen. In this way, research should focus not only on the effect of vaccination on transmission but also on the impact in mosquito biological processes such as oviposition and survival. Herein, we demonstrate that vaccination with an immunogenic and conserved peptide of the GT protein (GTp) reduced malaria vector survival suggesting its potential to be part of a multivalent vaccine for malaria control by reducing* Plasmodium* transmission.

## 2. Materials and Methods

### 2.1. Ethical Statement

All institutional (Instituto de Higiene e Medicina Tropical (IHMT) Ethical Committee and Divisão Geral de Alimentação e Veterinária (DAGV), Art° 8, Portaria number 1005/92 of 23rd of October), national (Decreto-Lei number 129/92), and European (Directive 86/609/EEC) guidelines for the care and use of laboratory animals were followed. Animal procedures were carried out under approval number 023357 of DAGV.

### 2.2. Sequence Analysis

The amino acid sequence of AGAP007752 protein was used to search for the orthologues using Blastp (https://www.vectorbase.org/blast).* A. darlingi*,* A. sinensis*, and* A. stephensi* amino acid sequences were considered as orthologous and its sequences were obtained from the VectorBase database (https://www.vectorbase.org/blast). All sequences were aligned with MAFFT (v7) [22] and all positions containing gaps were excluded with GUIDANCE2 [23]. Molecular Evolutionary Genetics Analysis (MEGA, version 6) software was used to obtain the best model to build the phylogenetic tree [21]. The Maximum Likelihood tree was constructed based on a bootstrapping method with 1000 replicates, LG model, and a proportion of gamma distributed sites [20]. The tree generated was visualized and edited using FigTree v1.4.3 [24].

### 2.3. Prediction of Antigenic Determinants and Peptide Synthesis

The linear epitope of AGAP007752 sequence was determined with near 75% accuracy based on the prediction of antigenic determinants using the method developed by Kolaskar and Tongaonkar [25] available at http://imed.med.ucm.es/Tools/antigenic.pl. A putative antigenic GTp was selected to be synthetically produced by ProteoGenix SAS Company (Schiltigheim, France) for the immunoassays and vaccine formulation.

### 2.4. Consensus Sequence Peptides

To check for similarity between the antigenic epitope of* A. gambiae* GT protein, previously selected, and the orthologue* A. stephensi* protein (ASTE006385), the ClustalW program (http://europepmc.org/abstract/MED/21988835) at the EBI website (http://www.ebi.ac.uk/Tools/msa/clustalo/) was used to align the amino acid sequences.

### 2.5. Vaccine Formulations

Lyophilized antigen with a positive net charge (pH = 8.92) was reconstituted in 750 *μ*L of phosphate-buffered saline (PBS) and 50 *μ*L of acetic acid 0.6% (v/v) at a concentration of approximately 3.5 *μ*g/*μ*l peptide. The peptide-based vaccine composition was mixed 1 : 1 with Montanide ISA 50 V2 adjuvant (Seppic, France), at a final peptide concentration of 25 *μ*g/*μ*l. A control was prepared as above but with PBS replacing the peptide.

### 2.6. Mice Vaccination and Infection Challenge

For immunization, twelve five-week-old female Balb/c mice were obtained from Charles River Laboratories and kept in the IHMT animal facilities under controlled conditions. Mice from control group (*N* = 6) were primed and boosted intraperitoneally five times with 0.1 ml each containing adjuvant vaccine, every fortnight. A vaccinated group (*N* = 6) was immunized with the GTp-formulated vaccine. Four days after the last immunization, mice were infected with 10^7^* P. berghei* ANKA parasitized red blood cells by intraperitoneal inoculation or left uninfected as control.

### 2.7. Antibody Titer Determination

Before each immunization and four days after the last immunization, mouse tail blood was collected to obtain sera for determination of anti-GTp antibodies titers by ELISA.

A high binding 96-well ELISA plate (Costar®, MA, USA) was coated with 0.1 *μ*g of GTp protein diluted in 100 *μ*l PBS and incubated overnight at 4°C. The plate was then washed five times with Tris buffered saline (25 mM Tris HCl, 150 mM NaCl, and 2 mM KCl) containing 0.05% (v/v) Tween 20 (TBST), blocked with 300 *μ*l of 5% (w/v) milk (Bio-Rad, Hercules, CA, USA) at room temperature (RT) for 90 minutes and washed five times with TBST. For one hour at 37°C, serum samples were incubated, and, after washing the plate, the secondary anti-mouse AP-conjugated immunoglobulins (Sigma-Aldrich, St. Louis, Missouri, USA) diluted 1 : 5,000 in TBST were added to each well for an incubation of one hour at 37°C. Washed plates were incubated with 1 mg/ml of p-nitrophenyl phosphate in substrate buffer (100 mM glycine, 1 mM MgCl2, 1 mM ZnCl2, and pH 10.4) at RT in the dark. Plates were then read at a wavelength of 405 nm in an ELISA plate reader (Triad Series Multimode Detector, Dynex Technologies, Chantilly, VA, USA) with Concert-Triad Series software (version 2.1.0.17) at a wavelength 405 nm. Negative and positive controls were included in the plates. Antibody titers were determined at a 1 : 500 serum dilution and were compared between GTp-immunized and control mice by Mann–Whitney test (*P* < 0.05) (SPSS v24.0) [26].

### 2.8. Mosquito Rearing

*A. stephensi* mosquitoes (SDA-500 strain) were obtained from the IHMT insectary, reared at 20°C in 70% humidity, under a 12 h light/dark photoperiod, and fed ad libitum on a 10% glucose solution. For experiments, only mosquitoes aged between 3 and 5 days were used.

### 2.9. *Anopheles stephensi* Infection with* Plasmodium berghei*

Mice blood samples were collected from mouse tail to determine parasitemia, using light microscopy after staining with Hemacolor® kit (EMD Millipore, Germany). When the parasitaemia reached 10–20% and 4–6 exflagellations/field were observed, mice were anesthetized and used to feed and infect female mosquitoes (*N* = 300/mice). Unfed female mosquitoes were excluded, while fully engorged mosquitoes were maintained at 19–21°C and 80% humidity for* P. berghei* development.

After blood meal, mosquitoes were kept in the cage with egg cups for oviposition evaluation. To count the number of eggs within groups, images from egg cups were taken and analyzed using ImageJ 1.50i [27]. Mosquito survival for 18 days after feeding was also evaluated by counting the number of survived mosquitoes between groups. Results from mosquitoes fed on GTp-immunized and control mice were compared by Mann–Whitney test (*P* < 0.05) (SPSS v24.0) [26].

### 2.10. Determination of* Plasmodium* Infection

To check for rates in parasite infection, at day 18 after blood-meal, cold anaesthetized female mosquitoes were used and salivary glands dissected to perform RNA extraction. RNA was immediately extracted using GRS FullSample Purification Kit (GRiSP, Porto, Portugal), quantified using a ND-1000 Spectrophotometer (NanoDrop ND1000, Thermo Fisher Scientific, Waltham, MA), and stored at −80°C. Using three biological replicates of each condition (immunized or nonimmunized), total RNA (100 ng/*μ*L) was used to synthesize cDNA using the iScript™ cDNA Synthesis Kit (Bio-Rad, CA, USA). qPCR reactions of 10 *μ*l were performed in triplicate using IQ™ SYBR® Green Supermix kit (Bio-Rad, CA, USA) in a CFX96 Touch Real-Time PCR (Bio-Rad, CA, USA) thermocycler. The following conditions were used: an initial cycle of denaturation at 95°C for 10 min, followed by 45 cycles of 95°C for 15 seconds and 55°C for 45 seconds to amplify the target* Plasmodium 18s rRNA* gene. Fluorescence readings were taken at 62°C after each cycle and a melting curve (60–95°C) was performed. To determine the reaction efficiency, standard curves were constructed with 5-fold serial dilutions of cDNA synthetized from total RNA from a pool of samples. Relative expression levels mosquito samples were normalized using the* ribosomal protein S7* (60°C) as reference gene and gene expression was analyzed using the CFX Manager™ Software version 3.1 (Bio-Rad, CA, USA).

### 2.11. Immunofluorescence Assay

To evaluate the serum ability to detect the native antigen on* A. stephensi* salivary glands, immunofluorescence assays (IFA) were performed. Salivary glands were dissected and stored at 4°C in 4% (w/v) paraformaldehyde solution (Sigma-Aldrich, St. Louis, Missouri, USA) diluted in PBS. For immunolocalization, stored tissues were placed in glass slides (BioMérieux, France) and washed three times for 15 minutes with PBS. Salivary glands were subsequently permeabilized with 0.2% (v/v) Triton X-100 (USB, Cleveland, OH, USA) in PBS for 30 minutes at RT and washed with PBS (three times for 15 minutes) and a blocking solution of 3% (w/v) bovine serum albumin (BSA) (Sigma-Aldrich, St. Louis, Missouri, USA) in PBS was added for 30 min at RT. After a washing step, PBS, primary antibody from nonimmunized mice and primary antibody from immunized mice, diluted (1 : 100) in blocking solution, was applied to respective wells and the slides were incubated overnight at 4°C in the dark. The secondary antibody, Alexa Fluor® 647 (magenta) from rabbit anti-mouse IgG (H+L) (Molecular Probes, Thermo Fisher Scientific, Waltham, Massachusetts, United States), diluted (1 : 100) in blocking solution was incubated for one hour at RT. After three PBS washes, a drop of ProLong® Gold Antifade Reagent with 4′,6′-diamidino-2-phenylindole (DAPI) (Invitrogen, Carlsbad, CA, USA) was placed over the tissues and then slides were sealed with a coverslip. After mounting, slides were placed in a humid dark box until microscopy analysis to prevent drying and fluorescence fading.

Sections of salivary glands were examined and photographed under 400x original magnification of a Nikon eclipse 80i fluorescence microscope with Nikon DS-Ri1 camera (Nikon Europe, Amsterdam, Netherlands) and appropriate filters (GFP/DAPI/CY5). Images were saved as high quality TIFF files using NIS-ELEMENTS BR 3.2 (Nikon Europe, Amsterdam, Netherlands) software, and the fluorescence intensity was compared between sections by ImageJ 1.50i [27]. Experiments were conducted in parallel and the image acquisition parameters were the same.

## 3. Results and Discussion

### 3.1. Glucose Transporter in Anophelines

When searching for potential targets to develop a mosquito stage vaccine, receptors or transmembrane transporters are considered important molecules to interrupt malaria transmission by targeting parasite adhesion and/or invasion and/or maintenance in the mosquito tissues [28, 29].

To determine the potential of AGAP007752 as a new malaria control approach, we hypothesized that such gene modulation could occur in other vectors of malaria, such as* A. stephensi* and may interfere in* Plasmodium* infection and vector sustainability.

**Figure 1:**
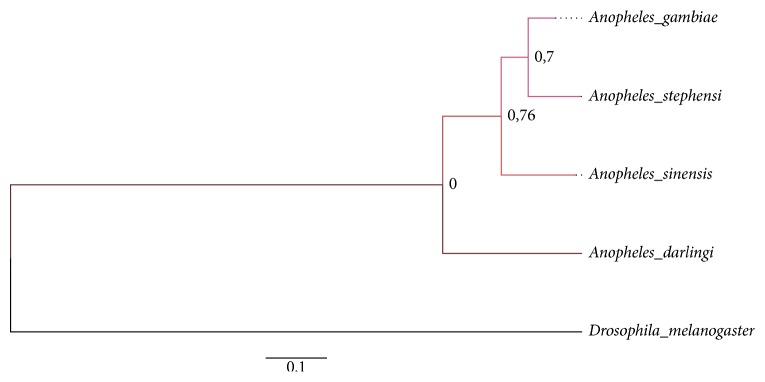
*Molecular Phylogenetic Analysis by Maximum Likelihood Method*. Using the Maximum Likelihood method based on the Le_Gascuel_2008 model [20], the tree with the highest log likelihood (−3359.935) is shown. A discrete Gamma distribution was used to model evolutionary rate differences among sites (5 categories (+G, parameter = 1.65)). The tree is drawn to scale, with branch lengths measured in the number of substitutions per site. The analysis involved 5 amino acid sequences with respective VectorBase accession number: AGAP007752:* Anopheles gambiae*, ASTE006385:* A. stephensi*, ASIS016443:* A. sinensis*, ADAC010569:* A. darlingi*, and CG15406:* Drosophila melanogaster* (outgroup). All positions containing gaps and missing data were eliminated and bootstrap values for internal branches are shown. Evolutionary analyses were conducted in MEGA6 [21].

Sequence alignment analysis demonstrates that the* A. gambiae* GT is closely related to the* A. stephensi* protein (ASTE006385) sharing 84% identical residues. Moreover, the* A. gambiae* amino acid sequence shares a high similarity with the* A. sinensis* (82%) and* A. darlingi* (70%). The evolutionary history of the AGAP007752 protein was inferred (Figure 1). These results highlight the high degree of conservation of GT between anophelines and the potential to use this target to block malaria transmission in different malaria vector species.

Both AGAP007752 and ASTE006385 proteins belong to the major facilitator superfamily responsible for the binding and transport of sugar molecules (InterPro: IPR005829 and IPR005828) [30]. And, as mentioned in previous studies, GT is a highly conserved transporter protein with 12 transmembrane domains and a unique N-linked oligosaccharide side-chain present in a large extracellular loop [17]. From all the predicted antigenic peptides of this protein, the longest sequence with high polarity (hydrophilic) and 80% identity with the* A. stephensi* protein (ASTE006385) and located between amino acids 190 and 260 (PDTPQTCLKKGRTAEAERSFMFYRGIRTQAEKTSALRQEFDNMEKFIEHNSGQNSRVTLADFKSREAKLGI) was selected for synthesis. The synthetic peptide with a molecular weight of 8.17 KDa and a purity of 94.5% was then used for immunization assays using* A. stephensi *mosquitoes.

### 3.2. Localization of the GT Conserved Peptide in* A. stephensi* during* P. berghei* Infection

**Figure 2:**
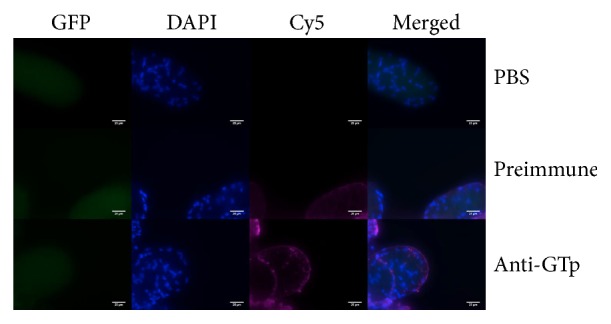
*Fluorescence Microscopy of A. stephensi-Infected Salivary Glands Triple-Stained*. Salivary glands were incubated with PBS, preimmune and anti-GTp immune serum. Green fluorescence corresponds to GFP-expressing* P. berghei*, blue to the nuclei stained with DAPI and magenta for Cy5-Alexa Fluor 647 to localize specific anti-GTp antibodies. The fluorescence intensity of SG Z-stack and merged images was compared and then analyzed in the same conditions. A specific and localized recognition was evident when SGs were incubated with the anti-GTp serum. Z-stack maximum intensity projections and a merge image of the three channels are given. Green: GFP-expressing* P. berghei*. Blue: DAPI-counterstaining of the nuclei. Magenta: Cy5-Alexa Fluor 647. Bar: 25 *µ*m.

An immunofluorescence assay was performed to characterize the presence and localization of GT in* A. stephensi* salivary glands during* Plasmodium* infection (Figure 2). As negative control, nucleated and infected SGs-cells were incubated with PBS and led to no reaction in Cy5 channel. When preimmune serum was added, a diffuse fluorescence was observed in the Z-stack images related to a nonspecific interaction between several proteins and the polyclonal serum. As observed in Figure 2, the fluorescence labeling is higher and specific on Cy5 filter with anti-GTp serum. These results confirm the specific interaction of antibodies with GTp in infected salivary glands of* A. stephensi*, distributed in the membrane heterogeneously.

These results corroborated the presence of GT in* A. stephensi* SGs, which is in accordance with previous reports of GT proteins as a key for parasite recognition in the salivary glands [14, 15], providing a regulated electrochemical gradient and nutrients to cell and parasite maintenance [12].

### 3.3. Impact of GTp Immunization on Mosquito Infestations and Parasite Infection

**Figure 3:**
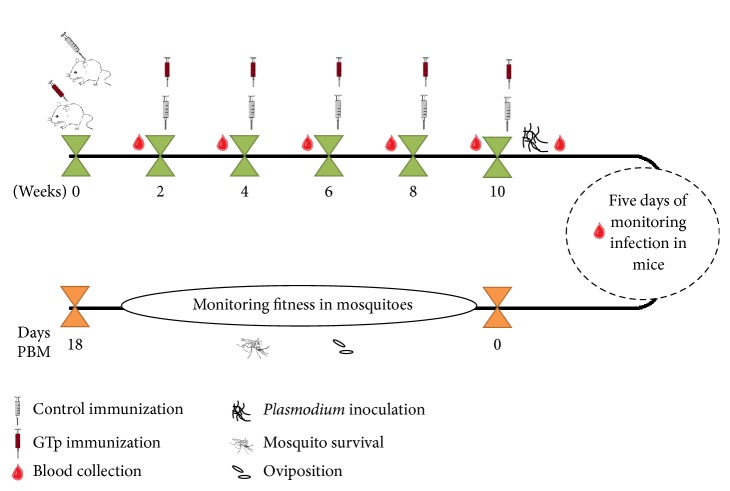
*Schematic Overview of Vaccination Trial*. From twelve five-week-old female Balb/c mice, six were primed with PBS and the others with GTp-formulated vaccine. Every fortnight, mice were boosted five times intraperitoneally. Four days after the last immunization, mice were infected with* P. berghei* parasitized red blood cells by intraperitoneal inoculation or left uninfected as control. Blood samples were collected to determinate antibody titers during immunization and after* Plasmodium* infection. For five days the infection was monitored and when the parasitaemia reached 10–20% and 4–6 exflagellations/field were observed, mice were anesthetized and used to feed and infect female mosquitoes (*N* = 300/mice). Mosquito survival and oviposition were assessed until the 18th day after blood meal (PBM).

Vaccination trials were conducted to elucidate the impact of GTp on mosquito oviposition and mortality and* P. berghei* parasite infection of salivary glands (Figure 3).

**Figure 4:**
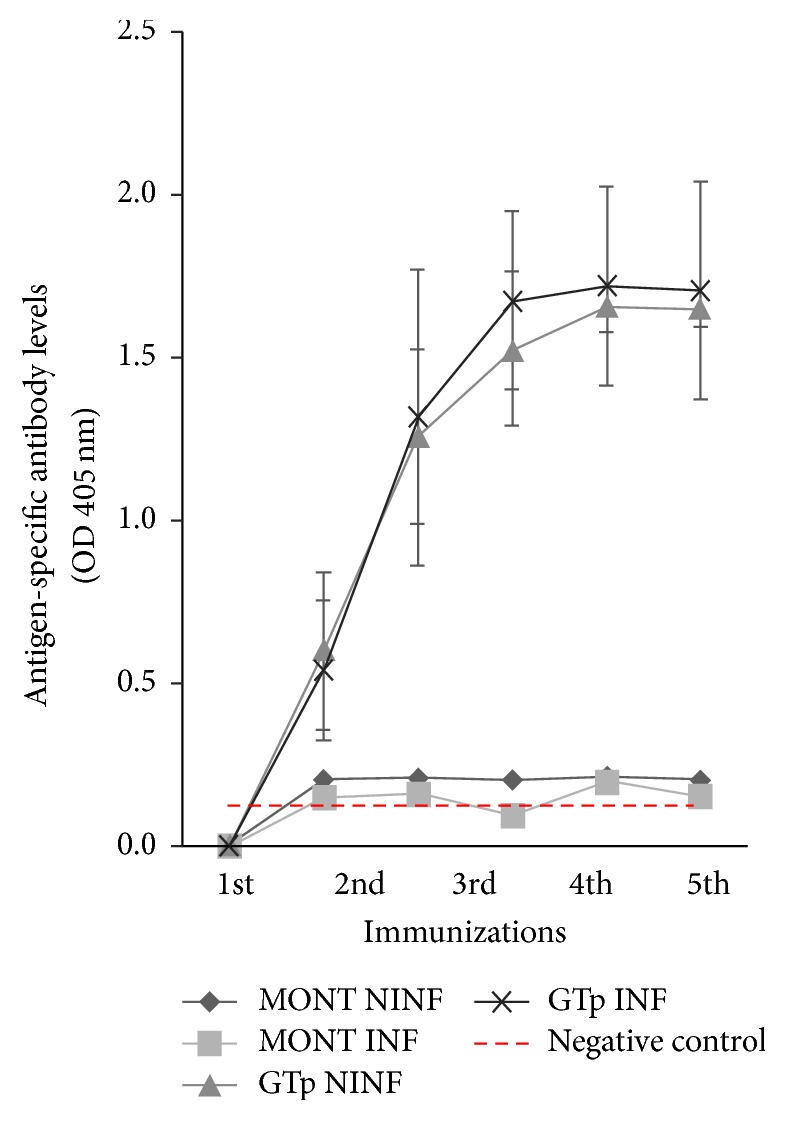
*Mice Immunization with GTp after Challenge or Not with P. berghei*. Comparison between GTp-immunized and control mice with and without* Plasmodium* infection. Antibody titers were determined by ELISA assays and* Plasmodium* inoculation is represented with a red arrow. Negative control is represented in a red dashed line. Statistical analyses were performed using Mann–Whitney test (*P* < 0.05). MONT NINF: control mice without* Plasmodium* infection; MONT INF: control mice with* Plasmodium* infection; GTp NINF: GTp-immunized mice without* Plasmodium* infection; GTp INF: GTp-immunized mice with* Plasmodium* infection.

During subsequent GTp immunizations, the antibody titers started to increase, even after pathogen inoculation in mice (Figure 4), revealing a successful immune response to GTp administration and no consequence in the parasite erythrocytic cycle, as expected for an* Anopheles*-specific antigen.

**Figure 5:**
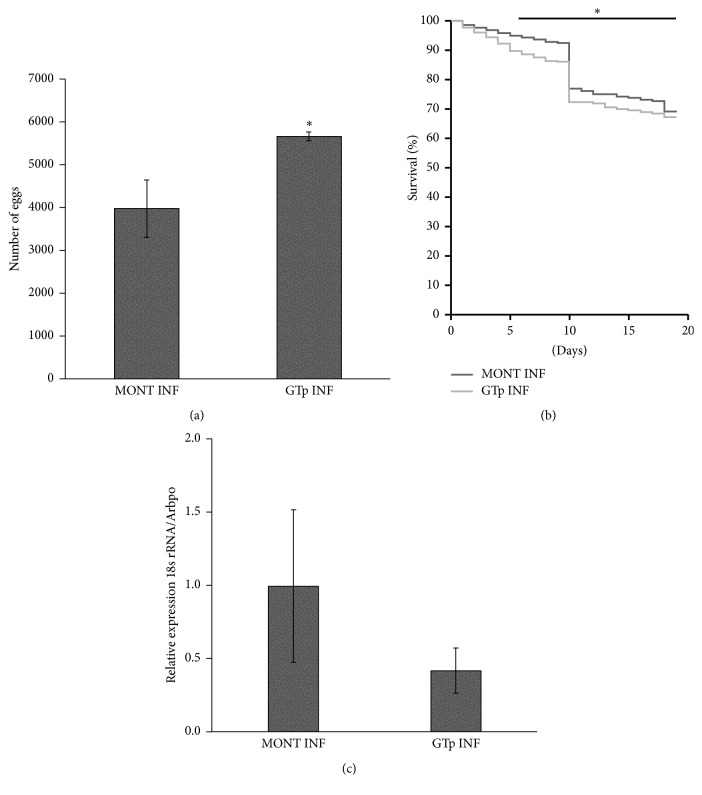
*The Effect of Anti-GTp Antibodies on A. stephensi Mosquitos and Malaria Parasites*. (a) Oviposition. Total number of eggs laid after each experiment (*N* = 3) were counted and compared between GTp-immunized and nonimmunized groups. (b) Mosquito survival after blood meal with anti-GTp immune serum and preimmune serum. (c).* Plasmodium* mRNA levels at 18th day after blood meal in SGs tissues. Statistical analyses of (a) and (b) results were performed using Mann–Whitney test and for (c) using CFX Manager Software (^*∗*^*P* < 0.05). MONT INF: control mice with* Plasmodium* infection; GTp INF: GTp-immunized mice with* Plasmodium* infection.

Immune-stimulated mice with or without* Plasmodium* infection were used for female* A. stephensi* mosquitoes blood meal containing* Plasmodium* parasites and antibodies against salivary GTp, and, then, the oviposition (Figure 5(a)), mosquito survival (Figure 5(b)), and parasite infection (Figure 5(c)) were assessed.

As expected, as a carbohydrate and precursor in the synthesis of glycogen, trehalose [31], and lipids [32], glucose is essential for insect development, metabolism, and oogenesis [33]. Therefore, disequilibrium in the levels of this energetic component could alter survival, mating, host seeking, and oviposition behaviors [34]. Besides, an impact on this pathway could slightly decrease* Plasmodium* infection and consequently improve the mosquito fitness.

Such imbalance probably happened during vaccination trials, where anti-GTp antibodies interacted with the target protein in* A. stephensi* salivary glands leading to a significant increase of about 29% in the number of the eggs (*P* = 0.05) (Figure 5(a)) and an average reduction of 4.67% in mosquito survival during all replicas with significant impact between the 5th and the 18th day (Log Rank/Mantel-Cox: *P* = 0.085; Breslow/Generalized Wilcoxon: *P* < 0.0001; Tarone-Ware: *P* = 0.003) (Figure 5(b)). Further studies could evaluate eggs viability to focus on the effect of vaccination in the quantity of glucose-metabolites inside the egg and subsequently egg resistance desiccation effect [35].

Regarding the interference of GTp in* Plasmodium*-infected SGs, our results point to a no statistical reduction in* P. berghei* mRNA levels in the* A. stephensi* tissue (Figure 5(c)). We believe that using higher amount of antibodies anti-GTp acquisition by mosquitoes or even immunizing mice with the full length protein would significantly increase the reduction of the infection. Moreover, posttranslational modifications like glycosylation, often occurring in GTs, cannot be ruled out as potential efficiency promoter on the design of vaccines by increasing protein immunogenicity.

## 4. Conclusions

To break the complex interplay during malaria parasite transmission, a mosquito stage vaccine that works in tandem with other interventions would be a critical tool for malaria elimination/eradication. GTp is a conserved and immunogenic peptide that reduces* A. stephensi* survival, therefore suggesting its potential for the control of different malaria vectors. Further researchs on developing GT-based vaccines need to be conducted to reduce mosquito survival and parasite infection/multiplication.
